# Surgical resection of recurrent gastrointestinal stromal tumor after interruption of long-term nilotinib therapy

**DOI:** 10.1186/s40792-016-0266-y

**Published:** 2016-11-19

**Authors:** Takahito Sugase, Tsuyoshi Takahashi, Takashi Ishikawa, Hiroshi Ichikawa, Tatsuo Kanda, Seiichi Hirota, Kiyokazu Nakajima, Koji Tanaka, Yasuhiro Miyazaki, Tomoki Makino, Yukinori Kurokawa, Makoto Yamasaki, Shuji Takiguchi, Toshifumi Wakai, Masaki Mori, Yuichiro Doki

**Affiliations:** 1 Department of Gastroenterological Surgery, Graduate School of Medicine, Osaka University, 2-2 Yamadaoka, Suita, Osaka 565-0871 Japan; 2 Division of Digestive and General Surgery, Graduate School of Medical and Dental Sciences, Niigata University, Niigata, Japan; 3 Department of Surgery, Sanjo General Hospital, Niigata, Japan; 4 Department of Surgical Pathology, Hyogo College of Medicine, Nishinomiya, Hyogo Japan

**Keywords:** Nilotinib, Resistant GIST, Secondary mutation

## Abstract

**Background:**

Nilotinib inhibits the tyrosine kinase activities of ABL1/BCR-ABL1, KIT, and platelet-derived growth factor receptors (PDGFRs). The results of a phase III clinical trial indicated that nilotinib could not be recommended for broad use as first-line therapy for gastrointestinal stromal tumor (GIST). However, some clinical studies have reported the effectiveness of nilotinib. We report here the cases of two patients who underwent surgical resections of nilotinib-resistant lesions after long-term nilotinib administration.

**Case presentation:**

Two Japanese female patients, aged 66 and 70 years, experienced peritoneal recurrence of intestinal GIST several years after surgery. Both were registered in the ENESTg1 trial and received nilotinib therapy. Although they continued nilotinib administration with a partial response according to the protocol, nilotinib-resistant lesions, which were diagnosed as focally progressive disease, developed and complete surgical resection was performed. Pathological examination revealed that the tumors were composed of viable KIT-positive spindle cells, and the recurrent tumors were diagnosed as nilotinib-resistant GIST. In gene mutation analysis, a secondary *KIT* gene mutation was detected in one case. Both patients have survived more than 5 years after the first surgery.

**Conclusions:**

Of patients who were registered in this trial, we have encountered two patients with long-term effects after nilotinib administration. Moreover, secondary mutations in the *KIT* gene, similar to those involved in resistance to imatinib, might be involved in resistance to nilotinib.

## Background

Gastrointestinal stromal tumors (GISTs) are the most common mesenchymal neoplasms of the gastrointestinal tract [1–4]. In 2000, imatinib, a selective tyrosine kinase inhibitor (TKI), was introduced for GIST therapy [5]. Since then, the prognoses of patients with unresectable and metastatic GIST have dramatically improved [6–9]. Worldwide, imatinib is the standard first-line therapy for patients with GISTs that are metastatic, unresectable, or both. However, despite such high clinical efficacy, imatinib treatment cannot achieve complete disease control. Nearly 90% of patients undergoing imatinib therapy experience disease progression following a significant response despite treatment continuation. Various treatments are available for patients with secondary imatinib resistance. In cases with limited progression wherein there are few lesions with a limited distribution, local therapies, including surgical resection, have been effective [10–12]. Moreover, gene mutation analysis revealed that lesions acquired resistance via secondary *KIT* mutations in addition to primary *KIT* mutations. Acquired resistance to imatinib is most commonly caused by secondary *KIT* mutations in other exons that arise during tyrosine kinase inhibitor therapy [6, 13–17].

Nilotinib is a selective tyrosine kinase inhibitor that targets ABL1, BCR-ABL, KIT, PDGFRα and PDGFRβ, and DDR-1 and DDR-2. Nilotinib has in vitro inhibitory activity similar to that of imatinib against KIT and platelet-derived growth factor receptors (PDGFRs) [18–20]. A phase III trial (ENESTg1) was performed to clarify the efficacy and safety of nilotinib compared to imatinib as first-line therapy for patients with advanced GISTs. In these trial results, although tolerance to nilotinib was similar to that of imatinib, nilotinib treatment failed to show superiority based on the primary end point of progression free-survival [21]. Because of this, nilotinib could not replace imatinib as first-line therapy for metastatic GIST. However, we have encountered two patients who have experienced long-term effects after nilotinib administration in the ENESTg1 trial and showed focal resistance. We resected each resistant lesion and continued molecular targeting therapy.

In this report, we assessed the therapeutic strategy and mechanism of nilotinib resistance.

## Case presentation

### Patient 1

**Fig. 1:**
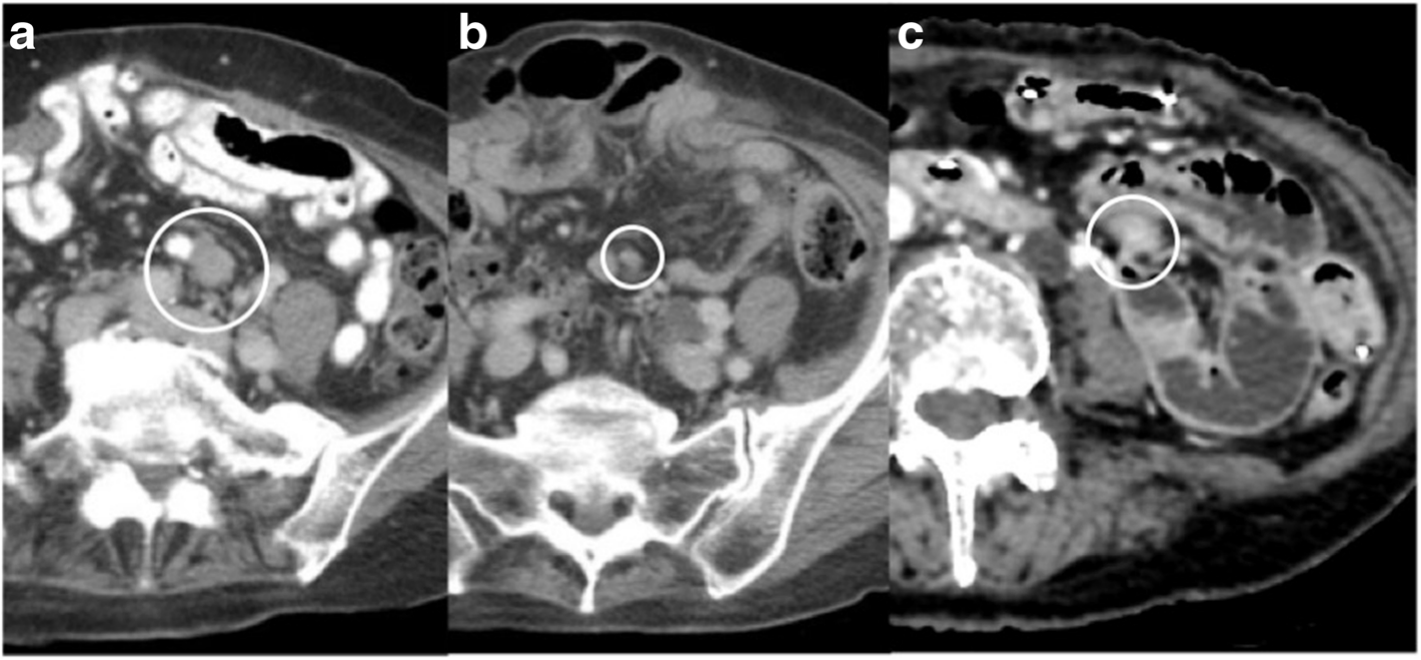
Case 1 imaging findings. **a** Abdominal CT at study enrollment. **b** Abdominal CT 3 months after start of nilotinib therapy. **c** Abdominal CT of the developing nilotinib-resistant tumor

A 76-year-old woman was diagnosed with a small intestinal primary GIST and underwent partial jejunum resection via open surgery. The tumor stained positively for CD117 (KIT) and CD34, and it was composed of spindle cells with >5 mitoses/50 high-power fields (HPF). Gene mutation analysis revealed a Lys (AAG) 558 to Asn&Pro (AACCCG) *KIT* mutation in exon 11. Postoperatively, she was followed-up strictly without adjuvant therapy. Two years after operation, a 15-mm peritoneal metastasis was discovered in the mesentery (Fig. 1a). We informed her of the randomized phase III trial (ENESTg1), and she agreed to enroll in the trial. After assignment to the nilotinib arm, she was treated with nilotinib. Because of several adverse events, including grade 2 appetite loss and skin bruising, she continued this treatment for 57 months at a decreased nilotinib dose according to the protocol guidelines and achieved a partial response (Fig. 1b).

**Fig. 2:**
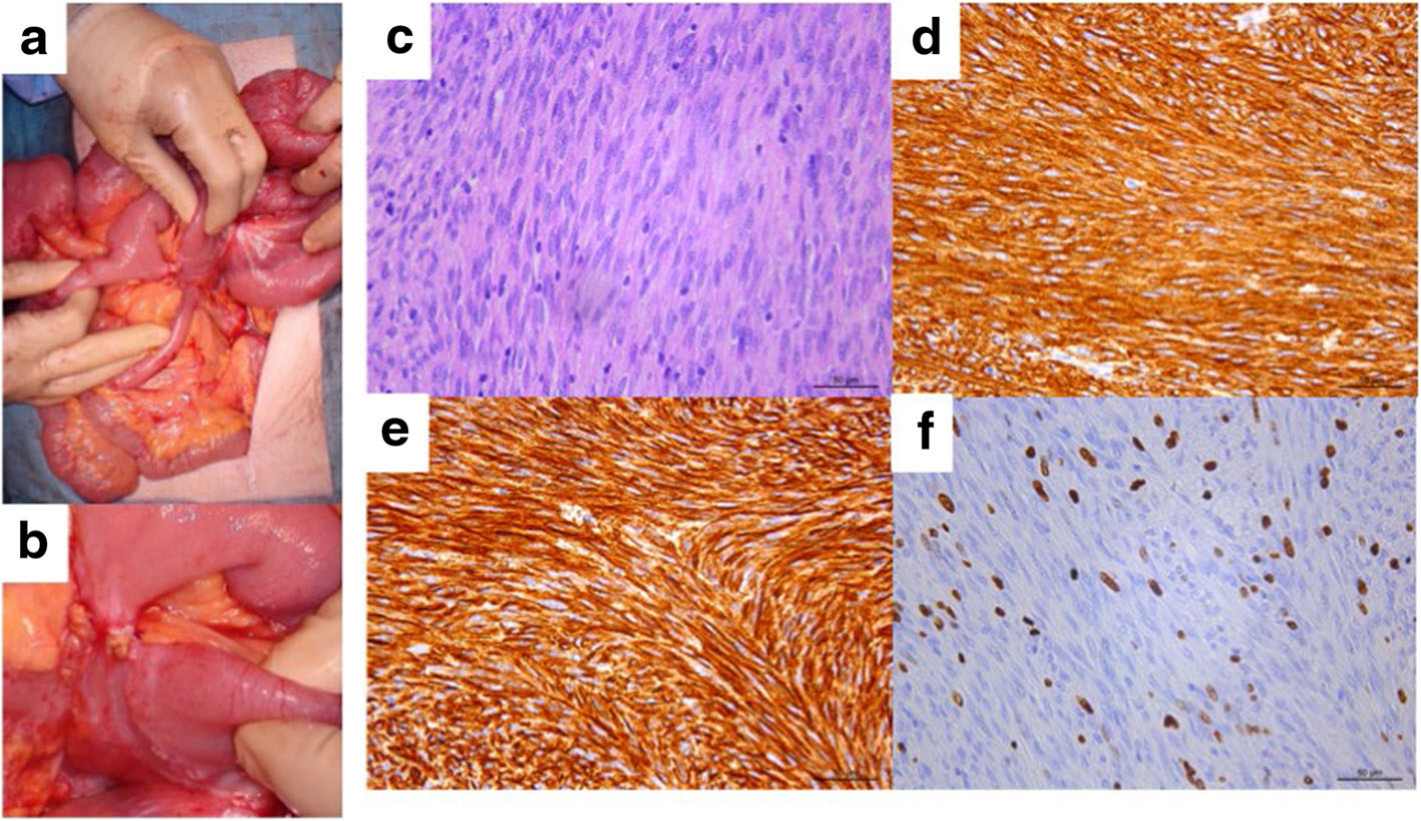
Case 1 surgical and pathological images. **a**, **b** Intraoperative photography. **c** Hematoxylin and eosin staining (×400). **d**–**f** Immunohistochemical staining of KIT/CD117 (**d**), CD34 (**e**), and MIB-1 (**f**) (×400)

Fifty-seven months after nilotinib administration, she experienced abdominal distention and vomiting. From imaging examinations, she was diagnosed with ileus due to a recurrent tumor (Fig. 1c). Since we diagnosed her with focal resistance, she underwent surgical tumor resection (Fig. 2a and b). Pathological examination revealed that the tumor was composed of viable spindle cells with 15 mitoses/50 HPF that stained positively for CD117 (KIT) and CD34 (Fig. 2c–f). From the above findings, we diagnosed the patient with recurrent nilotinib-resistant GIST. According to gene mutation analysis, the resistant GIST contained the same genetic *KIT* mutation in exon 11 observed in the primary GIST without any secondary mutations. After an additional surgery, nilotinib administration has been continued for 21 months, with no evidence of recurrence.

### Patient 2

**Fig. 3:**
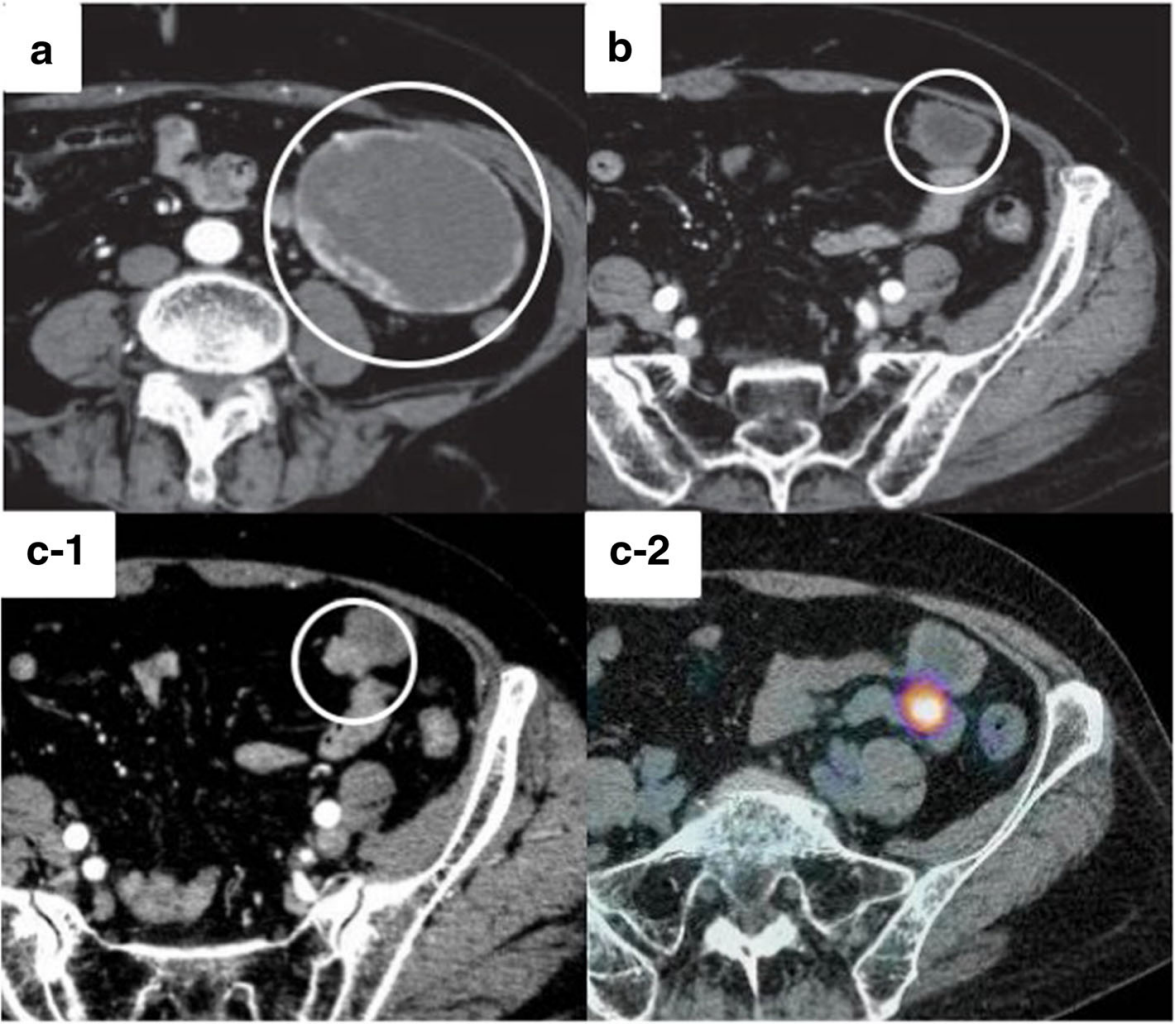
Case 2 imaging findings. **a** Abdominal CT at study enrollment. **b** Abdominal CT 3 months after start of nilotinib therapy. **c** Abdominal CT (C-1) and PET-CT (C-2) of the developing nilotinib-resistant tumor

Similar to the patient in case 1, a 66-year-old woman was diagnosed with a primary submucosal tumor in the small intestine and underwent partial jejunum resection via open surgery. The tumor stained positively for CD117 (KIT) and was composed of spindle cells with 19 mitoses/50 HPF. From the above findings, tumor was finally diagnosed as a GIST originating from the small intestine. Gene mutation analysis revealed a Del-5a.a (557-561) *KIT* mutation in exon 11. She received postoperative adjuvant imatinib (400 mg daily) for 1 year. As adverse events of imatinib, she experienced leukopenia (grade 3), thrombopenia (grade 2), and leg edema (grade 1). Four years after the operation, a 77-mm peritoneal metastasis was discovered (Fig. 3a). We informed her of the randomized phase III trial (ENESTg1), and she agreed to enroll. After assignment to the nilotinib arm, she was treated with nilotinib. She experienced no adverse events. She continued this treatment with a partial response for 41 months, according to the protocol guidelines (Fig. 3b).

**Fig. 4:**
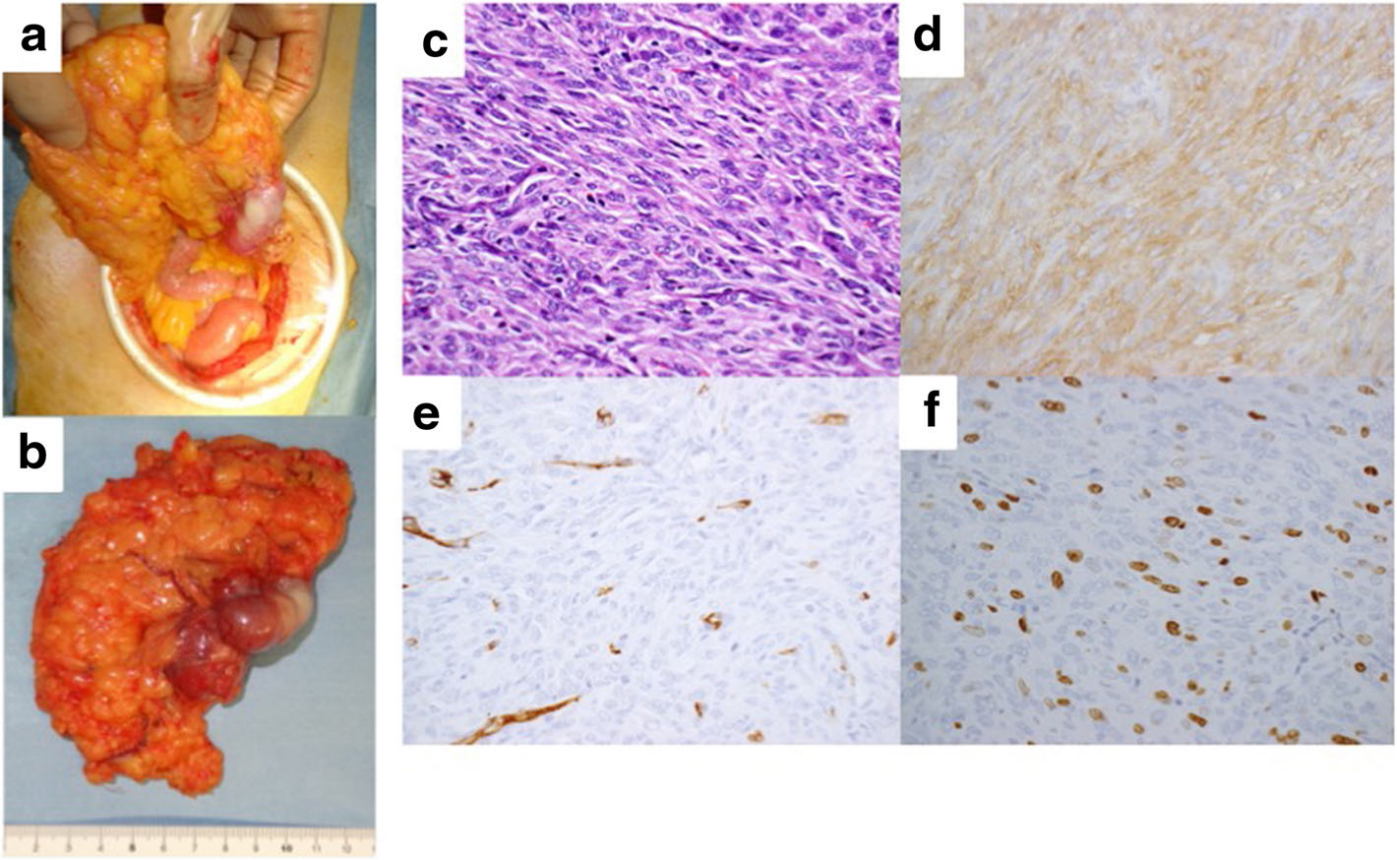
Case 2 surgical and pathological images. **a**, **b** Intraoperative photography. **c** Hematoxylin and eosin staining (×400). **d**–**f** Immunohistochemical staining of KIT/CD117 (**d**), CD34 (**e**), and MIB-1 (**f**) (×400).

Forty-one months after nilotinib administration, an abdominal computed tomography (CT) scan showed a 55-mm enhanced tumor area in the tumor margin, and fluorodeoxyglucose (FDG) accumulation was observed in the tumor margin by positron emission tomography-CT (PET-CT) (Fig. 3c). Because her tumor exhibited focal progression, she underwent surgical tumor resection (Fig. 4a and b). Pathological examination revealed a tumor composed of viable spindle cells with 34 mitoses/50 HPF and positive CD117 (KIT) staining (Fig. 4c–f). From the above findings, we diagnosed the patient with recurrent nilotinib-resistant GIST. According to gene mutation analysis, the resistant GIST contained not only the primary genetic *KIT* mutation in not only exon 11 but also secondary *KIT* mutation in exon 13 (Asn655Thr). After an additional surgery, since the nilotinib clinical trial was complete, we suggested that the patient receive imatinib therapy. However, the patient opted for observation only because of adverse events due to imatinib therapy. Sixteen months after reoperation, an abdominal CT scan showed multiple peritoneal metastases. Imatinib (300 mg daily) was administered for recurrence, and the patient now has stable disease (SD).

We summarized the main characteristics of two cases with nilotinib therapy in Table 1.

**Table 1:** Summary of two case of nilotinib resistant GIST

	Case 1	Case 2
Age (year)	70	66
Gender	Female	Female
Primary lesion	Small intestine	Small intestine
Modified-Fletcter’s classification	High risk	High risk
Primary KIT mutation	Exon11 Lys558 to Asn&Pro	Exon11 Del-5a.a.(557–561)
Adjuvant therapy	None	Imatinib (1 year)
Recurrence site	Peritoneal dessemination	Peritoneal dessemination
Duration from nilotinib administration to resistance (month)	56	53
Reason of resection for metastasis	Ileus for metastasis	Increase of metastasis
Secondary mutation	None	Exon13 Asn655Thr

## Discussion

Nilotinib is a selective tyrosine kinase inhibitor that targets ABL1, BCR-ABL1, KIT, PDGFRα and PDGFRβ, and DDR-1 and DDR-2. It has in vitro inhibitory activity similar to that of imatinib against KIT and PDGFRs [18–20]. For chronic myelogenous leukemia (CML), a phase III randomized controlled trial showed the benefits of first-line nilotinib treatment compared to imatinib [22]. Currently, nilotinib is a first-line treatment for CML according to the National Comprehensive Cancer Network (NCCN) guidelines [23]. Since the same effect as CML has been expected for GIST, clinical trials comparing nilotinib with imatinib have been performed. Previously, nilotinib was reported to be efficacious in imatinib- and/or sunitinib-resistant GIST [24–27]. The randomized multi-center phase III ENESTg1 trial was performed to compare imatinib and nilotinib as first-line treatment for distant metastasis or unresectable GIST [21]. In this trial, 647 people were registered, and patients demonstrated good tolerance to nilotinib compared with imatinib. However, nilotinib did not exhibit superiority in the progression-free survival (PFS) as the primary endpoint, with a PFS of 25.9 months in the nilotinib group vs. 29.7 months in the imatinib group. In addition, in subgroup analysis, patients with exon 11 mutations had better prognosis in overall survival or PFS both imatinib and nilotinib treatment group. In the present cases, the two patients with primary GISTs who participated in the ENESTg1 trial also had *KIT* mutations in exon 11, suggesting that long-term control of disease progression might be possible.

For patients with imatinib-resistant GIST, although sunitinib is recommended as treatment, surgery and continued imatinib therapy have been effective against focally progressive disease [11, 12, 28, 29]. Hasegawa et al. reported that patients with imatinib-resistant GISTs that are small, have few foci, and are of gastric origin might benefit from surgery plus continued imatinib therapy, with considerable tolerance and safety [30]. Additionally, in the two cases mentioned here, since the patients had good disease control after long-term nilotinib administration and no distant metastasis, similar to the therapeutic strategy for imatinib-resistant GIST, surgical resection was a good option. From pathological examination, we noted positive KIT staining, which was also similar to that seen in imatinib-resistant GIST. Patient 1, who underwent complete surgical resection and continued nilotinib therapy, has lived for 21 months with no recurrence.

In imatinib-resistant lesions, secondary *KIT* mutations were usually acquired in addition to primary *KIT* mutations [10–12]. Nishida et al. reported that 33 out of 45 tumors (73%) harbored secondary *KIT* mutations in the *KIT* kinase domain in GIST patients treated with imatinib therapy [13]. In case 2, in which the lesion developed resistance during nilotinib administration for recurrent GIST in addition to the primary *KIT* mutation in exon 11, the resistant lesion had a secondary *KIT* mutation in exon 13. Secondary mutations may lead to nilotinib resistance, similarly to those involved in imatinib resistance.

In GISTs showing secondary resistance to imatinib, a particular Val654Ala mutation has been detected as the exon 13-type secondary c-*kit* gene mutation. On the other hand, Asn655Thr mutation at exon 13 of the c-*kit* gene was observed as a secondary mutation in case 2 with secondary resistance to nilotinib. Kinoshita et al. reported that Asn655Lys mutation at exon 13 which was observed as a primary mutation in a sporadic GIST was imatinib-sensitive [31]. Since the codon number in Asn655Thr and Asn655Lys is the same, there is a possibility that Asn655Thr mutation in the present case 2 might be nilotinib-resistant but imatinib-sensitive. However, the properties of resistance to imatinib and nilotinib might be different from each other in Asn655Thr and Asn655Lys because the substituted amino acid is different between them. Effectiveness of imatinib in Asn655Thr remains to be clarified.

## Conclusions

The results of the ENESTg1 trial indicated that nilotinib cannot be recommended instead of imatinib for broad use as first-line treatment for patients with distant metastasis or unresectable GIST. Of patients who were registered in this trial, we have encountered two patients with long-term effects after nilotinib administration. Moreover, with regard to nilotinib resistance, secondary *KIT* mutations might be involved, similarly to those involved in imatinib resistance.

## Abbreviations

CML: Chronic myelogenous leukemia
GIST: Gastrointestinal stromal tumor
HPF: High power fields
NCCN: National comprehensive cancer network
PDGFRA: Platelet-derived growth factor receptor
PET-CT: Positron emission tomography-CT
PFS: Progression-free survival
SD: Stable disease
TKI: Tyrosine kinase inhibitor
